# Disparities in Patient Portal Engagement Among Patients With Hypertension Treated in Primary Care

**DOI:** 10.1001/jamanetworkopen.2024.11649

**Published:** 2024-05-15

**Authors:** Rasha Khatib, Nicole Glowacki, Eva Chang, Julie Lauffenburger, Mark J. Pletcher, Alvia Siddiqi

**Affiliations:** 1 Advocate Aurora Research Institute, Advocate Health, Milwaukee, Wisconsin; 2 Center for Healthcare Delivery Sciences (C4HDS), Department of Medicine, Brigham and Women’s Hospital and Harvard Medical School, Boston, Massachusetts; 3 Division of Pharmacoepidemiology and Pharmacoeconomics, Department of Medicine, Brigham and Women’s Hospital and Harvard Medical School, Boston, Massachusetts; 4 Department of Epidemiology and Biostatistics, University of California, San Francisco; 5 Enterprise Population Health, Advocate Health, Rolling Meadows, Illinois

## Abstract

**Importance:**

Patient portals are increasingly used for patient-clinician communication and to introduce interventions aimed at improving blood pressure control.

**Objective:**

To characterize patient portal use among patients with hypertension managed in primary care.

**Design, Settings, and Participants:**

This retrospective cohort study used electronic health records linked with patient portal log file data from a large, diverse Midwestern health care system. Patients with hypertension who had a primary care visit from January 1, 2021, to December 31, 2021, were included. The first visit in 2021 was considered the baseline visit; patient portal engagement was evaluated during the following year. Multivariate logistic regressions, presented as odds ratios (ORs) and 95% CIs, were used to evaluate associations between patient characteristics and patient portal engagement, adjusting for potential confounders.

**Exposures:**

Primary exposures included 4 sociodemographic factors routinely collected in the electronic health record: race and ethnicity, insurance, preferred language, and smoking status.

**Main Outcomes and Measures:**

Indicators of patient engagement with the patient portal included accessing the patient portal at least once, accessing the portal within 7 days of at least 50.0% of primary care physician (PCP) visits, frequent logins (<28 vs ≥28), messaging (<2 vs ≥2), and sharing home blood pressure readings.

**Results:**

Among 366 871 patients (mean [SD], 63.5 [12.6] years), 52.8% were female, 3.4% were Asian, 7.8% were Hispanic, 19.7% were non-Hispanic Black, 66.9% were non-Hispanic White, and 2.3% were of other race or ethnicity. During the 1-year study period starting in 2021, 70.5% accessed the patient portal at least once, 60.2% accessed around the time of their PCP visits, 35.7% accessed the portal frequently, 28.9% engaged in messaging, and 8.7% shared home blood pressure readings. Compared with White patients, non-Hispanic Black and Hispanic patients had lower odds of any access (Black: OR, 0.53; 95% CI, 0.52-0.54; Hispanic: OR, 0.66; 95% CI, 0.64-0.68), access around PCP visit time (Black: OR, 0.49; 95% CI, 0.48-0.50; Hispanic: OR, 0.62; 95% CI, 0.60-0.64), frequent access (Black: OR, 0.56; 95% CI, 0.55-0.57; Hispanic: OR, 0.71; 95% CI, 0.69-0.73), and messaging (Black: OR, 0.63; 95% CI, 0.61-0.64); Hispanic: OR, 0.71; 95% CI, 0.69-0.73).

**Conclusions and Relevance:**

This cohort study of patients with hypertension found clear sociodemographic disparities in patient portal engagement among those treated in primary care. Without special efforts to engage patients with portals, interventions that use patient portals to target hypertension may exacerbate disparities.

## Key Points

**Question:**

Are there disparities in engagement with patient portals among patients treated for hypertension in primary care?

**Findings:**

This cohort study of 366 871 patients found that patients with hypertension who are Black or Hispanic, who are uninsured, whose preferred language is not English, and who smoke are less likely to engage with patient portals.

**Meaning:**

These findings suggest that as health care systems use patient portals more frequently for hypertension-related interventions and programs, disadvantaged patient groups are being left behind, potentially exacerbating existing disparities in blood pressure control.

## Teaser

This cohort study of patients with hypertension characterizes patient portal access and use of hypertension-specific functionalities.

## Introduction

Hypertension is a major public health problem affecting close to half of the US population.^1^ Hypertension management requires regular follow-up appointments with the clinical care team to adjust medications until blood pressure is controlled and to monitor progress once control is reached.^2^ Ongoing communication outside clinic visits between patients and the care team is also essential to hypertension management, and electronic health record (EHR) patient portals hold great promise in facilitating these communications.^3,4,5^

Health care systems and care teams are increasingly relying on patient portals to deliver interventions.^6^ Patients with hypertension can use the patient portal to communicate with the care team about medication titration questions, review their after-visit summary, view their laboratory results, and more. These activities have been linked to more engaged patients and better management of their disease.^7,8,9^ Patient portals are used as a platform by the care team to provide evidence-based educational material to the patient.^9^ Patient portals can be used to collect patient-reported data, such as home blood pressure readings, to enhance disease management, which has been linked to improved blood pressure control and has been recommended in clinical practice guidelines.^10,11^ More recently, patient portals have been used to invite patients to participate in clinical trials, giving these patients the potential advantage of better care that is associated with clinical trial participation.^12^

Disadvantaged patient populations, including patients in racial and ethnic minority groups, patients who lack insurance, and patients whose preferred language is not English, are less likely to have their blood pressure under control.^13,14,15,16^ It is unclear how engaged disadvantaged patients are with the patient portal. Given the importance of ongoing asynchronous communications for patients with hypertension, it is crucial to characterize patient portal use to understand its adoption and reach as a platform for hypertension management. It is also important to monitor and evaluate the potential digital divide among disadvantaged patient populations who share the greatest burden of hypertension and its clinical consequences.^14^ With data from a large and diverse Midwestern health care system, we aim to characterize patient portal access, using multiple definitions, and the use of hypertension-specific functionalities, including messaging the care team and sharing home blood pressure readings.

## Methods

This retrospective cohort study followed the Strengthening the Reporting of Observational Studies in Epidemiology (STROBE) reporting guideline for cohort studies.^17^ The study was approved by the Advocate Aurora Health Institutional Review Board, which waived informed consent for this retrospective, deidentified data set because of minimal risk to patients.

### Study Setting, Data Sources, and Population

We used data from Advocate Health–Midwest, a large and diverse health care system serving Wisconsin’s urban and rural populations and Illinois’ metropolitan population. The patient portal was introduced to the system in 2012. All patients are automatically offered an activation code at no cost. The messaging function between patients and clinicians has been available in the patient portal since it was first introduced, and both patients and clinicians can initiate messages. The home blood pressure monitoring functionality was introduced in 2018. The care team must create and share a home blood pressure monitoring order with the patient before the patient can use the functionality to manually enter readings.

The analytic cohort included adult (aged ≥18 years) patients who have a documented hypertension diagnosis (using the *International Statistical Classification of Diseases and Related Health Problems, Tenth Revision* [*ICD-10*] code I10) and at least 1 visit in 2021 to 1 of 312 primary care sites (family medicine, internal medicine, or geriatrics) and at least 1 additional primary care visit before 2021. Sociodemographic and clinical data were extracted from the EHR at the baseline visit, defined as the first visit in 2021. Each patient was followed up for 1 year to evaluate patient portal activity.

### Main Independent Variables

Independent variables included patient sociodemographic and clinical characteristics. Race and ethnicity (self-reported in the EHR as 2 separate fields) were recategorized into 5 mutually exclusive race and ethnicity groups, including Asian, Hispanic or Latino, non-Hispanic Black (Black or African American = yes, Hispanic or Latino = no or unknown), non-Hispanic White (White = yes, Hispanic or Latino = no), and other (American Indian or Alaska Native, Native Hawaiian, other Pacific Islander, or unknown). Patients who self-reported being Hispanic or Latino were categorized as such regardless of their race. Only 9042 patients (2.5%) had unknown race, and 7880 patients (2.2%) had unknown ethnicity, resulting in 5028 patients (1.4%) missing data for the combined race and ethnicity variable. Preferred language was categorized as English or non-English. Insurance status was categorized as commercial, Medicare, Medicaid, or self-pay. Tobacco use, which is a health behavior associated with disadvantaged patients and patients with social needs,^18^ was defined as current vs never or former.

Clinical characteristics included depression and diabetes identified using *ICD-10* codes. Uncontrolled baseline blood pressure was defined as a systolic blood pressure of 140 mm Hg or higher or a diastolic blood pressure of 90 mm Hg or higher at the baseline visit. Blood pressure–lowering medications were grouped by class and categorized into at least 2 medications currently on the active medication list vs fewer than 2 medications.^2^ The number of primary care physician (PCP) visits was defined as clinical encounters at internal medicine, family practice, or geriatrics departments and was categorized as 1 to 2 visits vs 3 or more, per clinical practice guideline recommendations.^19^

### Outcomes

Five indicators for patient portal engagement were created. Access was defined by the patient having logged into their patient portal account at least once during the 1-year follow-up. Frequency of access was also presented as medians (IQRs). The number of patient logins was winsorized at the 95th percentile.^20,21^ Accessed around PCP visit times was defined as logging in up to 7 days before or after at least 50.0% of the patient’s PCP encounters during the 1-year study period. Frequent access was defined as having at least 28 logins during the 1-year study period. This cutoff was selected based on the median number of logins among patients who accessed the portal at least once. Messaging was defined as sending more than 1 free-text secure message to the care team during the 1-year study period via the patient portal messaging functionality. This function has been reported in the literature as a proxy for more active patient engagement given the required involvement in sending messages.^22,23^ Sharing home blood pressure readings was evaluated among the subgroup of patients who received an self-measured blood pressure order from their care team and was defined as sharing at least 1 reading. This functionality allows the care team to create an order for the patient to manually enter discrete systolic and diastolic readings from their home to be shared with the care team.

### Statistical Analysis

Descriptive statistics are reported as numbers (percentages) for categorical or ordinal variables and means (SDs) or medians (IQRs) for continuous variables. Five multivariable logistic regression models were created to evaluate associations between patient characteristics and each of the 5 patient portal engagement indicators. All models included the same independent variables (age, sex, race and ethnicity, insurance, primary language, diabetes, tobacco use, depression, uncontrolled baseline blood pressure, blood pressure–lowering medications, and number of PCP visits) and presented as adjusted odds ratios (ORs) and 95% CIs. The analysis used ORs instead of relative risks even though the outcomes are not expected to be rare; ORs may slightly overestimate relative risks but are commonly reported in the literature, and logistic regression models may be more familiar to readers.^24^ Given the low proportions of missing data from the EHRs used in this study, no imputation of missing data was performed. Instead, patients missing any of the baseline visit variables included in the regression models were excluded from the cohort (93 patients [<0.1%]). A 2-tailed *P* < .05 was considered statistically significant. Analyses were completed using SAS software, version 9.4 (SAS Institute Inc).

## Results

### Description of Study Population

A total of 366 871 patients (mean [SD], 63.5 [12.6] years) with hypertension were included in the analysis (eAppendix in Supplement 1). Of these patients, 52.8% were female and 47.2% were male, 3.4% were Asian, 7.8% were Hispanic, 19.7% were non-Hispanic Black, 66.9% were non-Hispanic White, and 2.3% were of other races and ethnicities. Blood pressure during the baseline visit was controlled among 83.3% of patients, 28.1% were prescribed at least 1 blood pressure–lowering medication, and 59.1% had 3 or more PCP visits during the 1-year study period (Table 1).

**Table 1.** Characteristics of Patients With Hypertension Managed in Primary Care

Characteristic	No. (%) of patients		
	All patients	Did not access patient portals	Accessed patient portals
Total patients	366 871 (100.0)	108 332 (29.5)	258 539 (70.5)
Age group, y			
18-44	33 259 (9.1)	4876 (4.5)	28 383 (11.0)
45-64	139 166 (37.9)	34 001 (31.4)	105 165 (40.7)
65-74	122 811 (33.5)	37 967 (35.1)	84 844 (32.8)
≥75	71 635 (19.5)	31 488 (29.1)	40 147 (15.5)
Sex			
Male	173 191 (47.2)	54 269 (50.1)	118 922 (46.0)
Female	193 680 (52.8)	54 063 (49.9)	139 617 (54.0)
Race and ethnicity			
Asian	12 412 (3.4)	3407 (3.1)	9005 (3.5)
Hispanic	28 697 (7.8)	10 594 (9.8)	18 103 (7.0)
Non-Hispanic Black	72 119 (19.7)	26 431 (24.4)	45 688 (17.7)
Non-Hispanic White	245 306 (66.9)	65 106 (60.1)	180 200 (69.7)
Other^a^	8337 (2.3)	2794 (2.6)	5543 (2.1)
Insurance			
Commercial	153 500 (41.8)	31 093 (28.7)	122 407 (47.4)
Medicare	183 345 (50.0)	68 244 (63.0)	115 101 (44.5)
Medicaid	25 433 (6.9)	8995 (8.3)	16 438 (6.4)
Self-pay	4593 (1.3)	0	4593 (1.8)
Preferred language			
English	351 303 (95.8)	100 341 (92.6)	250 962 (97.1)
Non-English	15 568 (4.2)	7991 (7.4)	7577 (2.9)
Tobacco use			
No	322 205 (87.8)	90 350 (83.4)	231 855 (89.7)
Yes	44 666 (12.2)	7982 (16.6)	26 684 (10.3)
Diabetes			
No	247 850 (67.6)	69 080 (63.8)	178 770 (69.2)
Yes	119 021 (32.4)	39 252 (36.2)	79 769 (30.9)
Depression			
No	302 699 (82.5)	89 440 (82.6)	213 259 (82.5)
Yes	64 172 (17.5)	18 892 (17.4)	45 280 (17.5)
Prescribed ≥2 BP medications			
No	263 920 (71.9)	75 230 (69.4)	188 690 (73.0)
Yes	102 951 (28.1)	33 102 (30.6)	69 849 (27.0)
Uncontrolled baseline BP			
No	305 650 (83.3)	88 859 (82.0)	216 791 (83.9)
Yes	61 221 (16.7)	19 473 (18.0)	41 748 (16.2)
No. of PCP visits			
1-2	150 126 (40.9)	49 531 (45.7)	100 595 (38.9)
≥3	216 745 (59.1)	58 801 (54.3)	157 944 (61.1)

Abbreviations: BP, blood pressure; PCP, primary care physician.

^a^ Other includes American Indian or Alaska Native, Native Hawaiian, other Pacific Islander, and unknown.

### Overall Engagement With the Patient Portal

**Figure 1.**
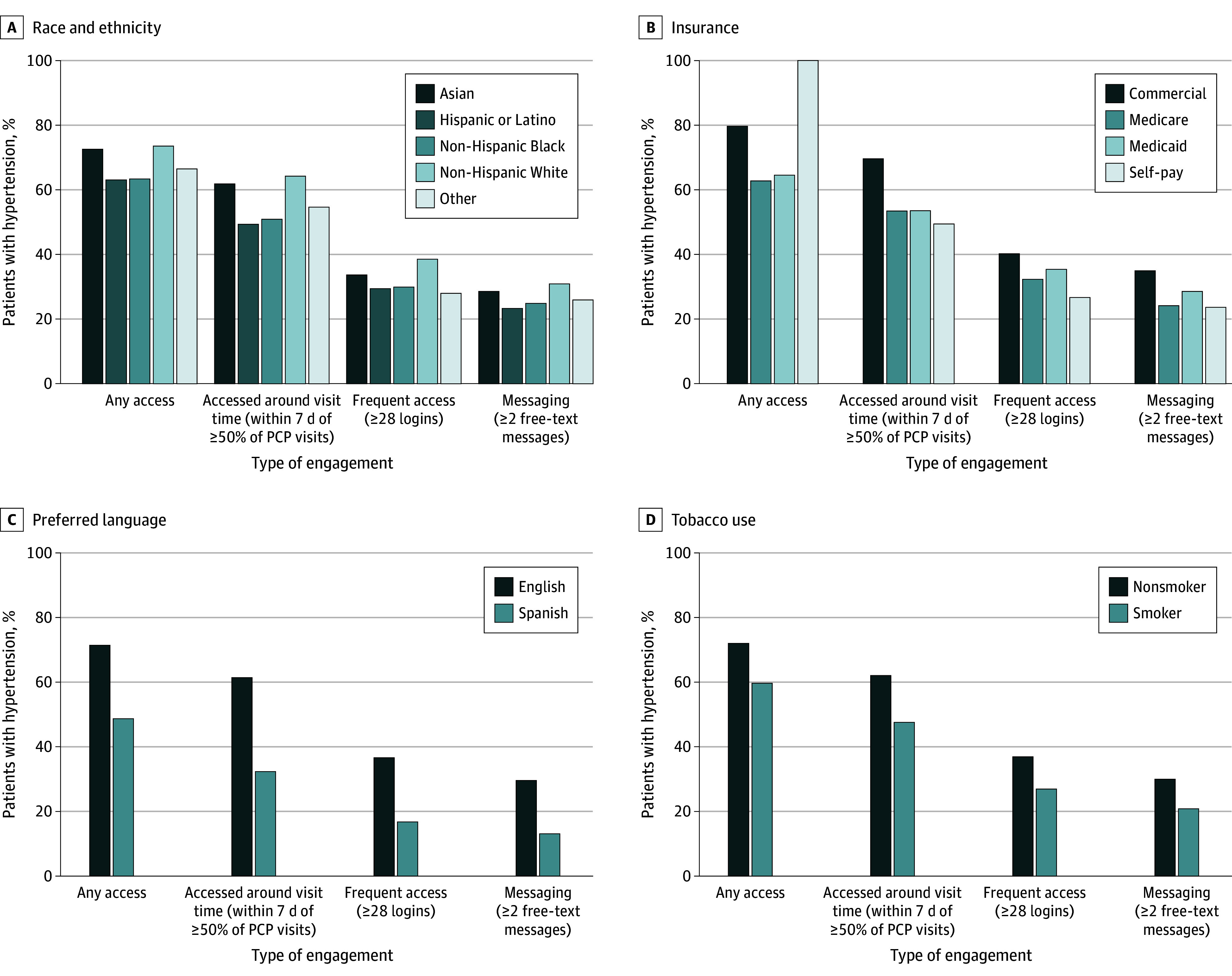
Patient Portal Engagement by Characteristics of the 366 871 Study Patients PCP indicates primary care physician.

**Figure 2.**
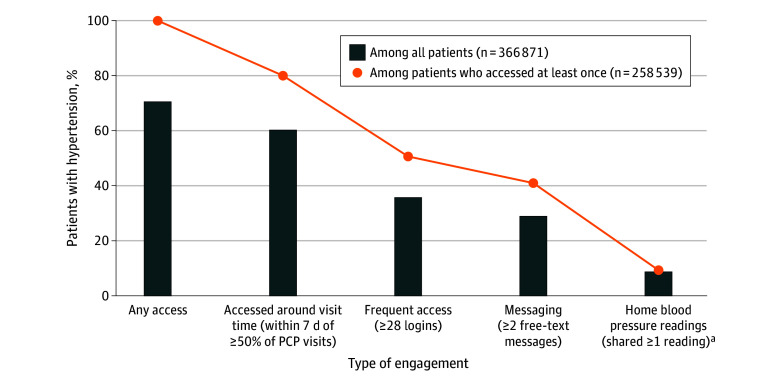
Proportion of Patients Who Engaged With the Patient Portal PCP indicates primary care physician. ^a^Limited to 553 patients who received an order from their care team to share home blood pressure (BP) readings via the patient portal.

The median (IQR) number of patient portal logins was 28.0 (10.0-65.0) among active users. On average, patients logged in a mean (SD) of 46.7 (49.8) times during the year. Figure 1 presents the proportion of patient portal engagement by patient sociodemographic characteristics among all patients. During the 1-year study, 70.5% of patients accessed the patient portal at least once, 60.2% accessed it around PCP visit time, 35.7% accessed frequently, and 28.9% engaged in messaging. Only 553 patients (<1.0%) received an order for home blood pressure monitoring; among them, 48 (8.7%) engaged in sharing at least 1 reading with the care team (Figure 2).

### Associations With Sociodemographic Characteristics

Adjusted analyses indicate clear differences in the sociodemographic characteristics of patients with hypertension who engaged with the patient portal (Table 2). Compared with White patients, non-Hispanic Black and Hispanic patients had lower odds of any access (non-Hispanic Black: OR, 0.53; 95% CI, 0.52-0.54; Hispanic: OR, 0.66; 95% CI, 0.64-0.68), access around PCP visit time (non-Hispanic Black: OR, 0.49; 95% CI, 0.48-0.50; Hispanic: OR, 0.62; 95% CI, 0.60-0.64), frequent access (non-Hispanic Black: OR, 0.56; 95% CI, 0.55-0.57; Hispanic: OR, 0.71; 95% CI, 0.69-0.73), and messaging (non-Hispanic Black: OR, 0.63; 95% CI, 0.61-0.64; Hispanic: OR, 0.71; 95% CI, 0.69-0.73). All 4593 patients in the self-pay insurance category accessed the patient portal at least once but had lower odds of access around PCP visit time (OR, 0.44; 95% CI, 0.42-0.47), frequent access (OR, 0.50; 95% CI, 0.46-0.53), and messaging (OR, 0.57; 95% CI, 0.53-0.61), compared with patients with commercial insurance. Similar trends with lower odds of engagement were observed among Medicare and Medicaid patients compared with commercially insured patients. Patients whose primary language is not English had lower odds of any access (OR, 0.40; 95% CI, 0.38-0.41), access around PCP visit time (OR, 0.35; 95% CI, 0.33-0.36), frequent access (OR, 0.37; 95% CI, 0.35-0.39), and messaging (OR, 0.40; 95% CI, 0.38-0.42) compared with patients whose preferred language is English. Tobacco users also had lower odds of access (OR, 0.50; 95% CI, 0.49-0.51), access around PCP visit time (OR, 0.50; 95% CI, 0.49-0.51), frequent access (OR, 0.58; 95% CI, 0.56-0.59), and messaging (OR, 0.55; 95% CI, 0.54-0.57) compared with non–tobacco users (Table 2). Associations between patient sociodemographic characteristics and receiving an order for and engaging with home blood pressure monitoring showed similar trends with differences in terms of age, race and ethnicity, insurance, preferred language, and tobacco use, although not all these associations were statistically significant (Table 3).

**Table 2.** Associations Between Patient Characteristics and Patient Engagement With the Patient Portal Among 366 871 Patients With Hypertension^a^

Independent variable	Any access (n = 258 539)		Accessed within 7 d of PCP visit (n = 206 947)		Frequent access (n = 131 034)		Messaging (n = 106 034)	
	No. (%) of patients	OR (95% CI)	No. (%) of patients	OR (95% CI)	No. (%) of patients	OR (95% CI)	No. (%) of patients	OR (95% CI)
Age group, y								
18-44	28 383 (85.3)	1 [Reference]	24 024 (75.2)	1 [Reference]	15 912 (47.8)	1 [Reference]	13 743 (41.3)	1 [Reference]
45-64	105 165 (75.6)	0.47 (0.45-0.49)	83 978 (64.1)	0.52 (0.50-0.53)	52 100 (37.4)	0.56 (0.54-0.57)	44 600 (32.1)	0.60 (0.59-0.62)
65-74	84 844 (69.1)	0.43 (0.41-0.44)	69 104 (60.1)	0.50 (0.48-0.52)	44 170 (36.0)	0.50 (0.48-0.51)	34 131 (27.8)	0.53 (0.51-0.55)
≥75	40 147 (56.0)	0.25 (0.24-0.26)	29 841 (45.3)	0.27 (0.26-0.28)	18 852 (26.3)	0.30 (0.29-0.31)	13 560 (18.9)	0.32 (0.31-0.33)
Sex								
Male	118 922 (68.7)	1 [Reference]	94 590 (58.1)	1 [Reference]	55 367 (32.0)	1 [Reference]	43 904 (25.4)	1 [Reference]
Female	139 617 (72.1)	1.34 (1.32-1.37)	112 357 (62.1)	1.33 (1.31-1.35)	75 667 (39.1)	1.45 (1.43-1.47)	62 130 (32.1)	1.49 (1.46-1.51)
Race and ethnicity								
Asian	9005 (72.6)	1.03 (0.99-1.08)	7163 (61.8)	0.97 (0.93-1.01)	4186 (33.7)	0.84 (0.81-0.88)	3535 (28.5)	0.91 (0.87-0.95)
Hispanic	18 103 (63.1)	0.66 (0.64-0.68)	13 177 (49.3)	0.62 (0.60-0.64)	8425 (29.4)	0.71 (0.69-0.73)	6677 (23.3)	0.71 (0.69-0.73)
Non-Hispanic Black	45 688 (63.4)	0.53 (0.52-0.54)	33 789 (50.9)	0.49 (0.48-0.50)	21 572 (29.9)	0.56 (0.55-0.57)	17 946 (24.9)	0.63 (0.61-0.64)
Non-Hispanic White	180 200 (73.5)	1 [Reference]	148 519 (64.2)	1 [Reference]	94 515 (38.5)	1 [Reference]	75 721 (30.9)	1 [Reference]
Other^b^	5543 (66.5)	0.67 (0.64-0.71)	4299 (54.7)	0.65 (0.62-0.68)	2336 (28.0)	0.62 (0.59-0.65)	2155 (25.9)	0.77 (0.73-0.81)
Insurance								
Commercial	122 407 (79.7)	1 [Reference]	101 132 (69.6)	1 [Reference]	61 623 (40.2)	1 [Reference]	53 589 (34.9)	1 [Reference]
Medicare	115 101 (62.8)	0.54 (0.53-0.56)	90 884 (53.4)	0.61 (0.59-0.62)	59 179 (32.3)	0.80 (0.78-0.82)	44 117 (24.1)	0.71 (0.69-0.73)
Medicaid	16 438 (64.6)	0.52 (0.51-0.54)	12 750 (53.5)	0.58 (0.56-0.60)	9004 (35.4)	0.83 (0.81-0.86)	7242 (28.5)	0.76 (0.74-0.78)
Self-pay	4593 (100.0)	NA^c^	2181 (49.4)	0.44 (0.42-0.47)	1228 (26.7)	0.50 (0.46-0.53)	1086 (23.6)	0.57 (0.53-0.61)
Primary language								
English	250 962 (71.4)	1 [Reference]	202 272 (61.4)	1 [Reference]	128 434 (36.6)	1 [Reference]	103 990 (29.6)	1 [Reference]
Non-English	7577 (48.7)	0.40 (0.38-0.41)	4675 (32.3)	0.35 (0.33-0.36)	2600 (16.7)	0.37 (0.35-0.39)	2044 (13.1)	0.40 (0.38-0.42)
Tobacco use								
Never or former	231 855 (72.0)	1 [Reference]	186 996 (62.0)	1 [Reference]	119 023 (36.9)	1 [Reference]	96 757 (30.0)	1 [Reference]
Current	26 684 (59.7)	0.50 (0.49-0.51)	19 951 (47.5)	0.50 (0.49-0.51)	12 011 (26.9)	0.58 (0.56-0.59)	9277 (20.8)	0.55 (0.54-0.57)
Diabetes								
No	178 770 (72.1)	1 [Reference]	144 378 (61.8)	1 [Reference]	87 776 (35.4)	1 [Reference]	71 710 (28.9)	1 [Reference]
Yes	79 769 (67.0)	0.90 (0.89-0.92)	62 569 (56.7)	0.92 (0.91-0.94)	43 258 (36.3)	1.07 (1.06-1.10)	34 324 (28.8)	1.09 (1.07-1.10)
Depression								
No	213 259 (70.5)	1 [Reference]	171 235 (60.3)	1 [Reference]	104 683 (34.6)	1 [Reference]	84 426 (27.9)	1 [Reference]
Yes	45 280 (70.6)	0.96 (0.94-0.98)	35 712 (59.9)	0.91 (0.90-0.93)	26 351 (41.1)	1.15 (1.13-1.17)	21 608 (33.7)	1.19 (1.17-1.22)
Prescribed ≥2 BP medication								
No	188 690 (71.5)	1 [Reference]	151 635 (61.3)	1 [Reference]	94 262 (35.7)	1 [Reference]	78 036 (29.6)	1 [Reference]
Yes	69 849 (67.9)	0.92 (0.91-0.94)	55 312 (57.5)	0.91 (0.89-0.92)	36 772 (35.7)	1.02 (1.01-1.04)	27 998 (27.2)	0.93 (0.92-0.95)
Uncontrolled baseline BP								
No	216 791 (70.9)	1 [Reference]	174 156 (60.8)	1 [Reference]	111 191 (36.4)	1 [Reference]	89 205 (29.2)	1 [Reference]
Yes	41 748 (68.2)	0.89 (0.88-0.91)	32 791 (57.0)	0.89 (0.87-0.90)	19 843 (32.4)	0.89 (0.88-0.91)	16 829 (27.5)	0.96 (0.94-0.98)
No. of PCP visits								
1-2	100 595 (67.0)	1 [Reference]	83 892 (56.1)	1 [Reference]	38 246 (25.5)	1 [Reference]	34 848 (23.2)	1 [Reference]
≥3	157 944 (72.9)	1.59 (1.56-1.61)	123 055 (63.4)	1.63 (1.61-1.66)	92 788 (42.8)	2.48 (2.45-2.52)	71 186 (32.8)	1.82 (1.80-1.85)

Abbreviations: BP, blood pressure; NA, not applicable; OR, odds ratio; PCP, primary care physician.

^a^ Adjusted for age, sex, race and ethnicity, insurance, primary language, diabetes, tobacco use, depression, uncontrolled baseline BP, BP-lowering medications, and number of PCP visits.

^b^ Other includes American Indian or Alaska Native, Native Hawaiian, other Pacific Islander, or unknown.

^c^ All patients in the self-pay insurance category accessed the patient portal at least once.

**Table 3.** Associations Between Patient Characteristics and Home BP Reading Functionalities in the Patient Portal^a^

Independent variable	BP monitoring order (n = 553)^b^		Patients shared BP readings (n = 48)^c^	
	No. (%) of patients	OR (95% CI)	No. (%) of patients	OR (95% CI)
Age group, y				
18-44	128 (0.4)	1 [Reference]	15 (11.7)	1 [Reference]
45-64	246 (0.2)	0.48 (0.38-0.60)	21 (8.5)	0.60 (0.29-1.24)
65-74	136 (0.1)	0.41 (0.30-0.56)	8 (5.9)	0.24 (0.06-0.96)
≥75	43 (0.1)	0.24 (0.15-0.37)	4 (9.3)	0.33 (0.06-1.81)
Sex				
Male	238 (0.1)	1 [Reference]	24 (10.1)	1 [Reference]
Female	315 (0.2)	1.20 (1.01-1.42)	24 (7.6)	0.69 (0.37-1.29)
Race and ethnicity				
Asian	10 (0.1)	0.60 (0.45-0.80)	1 (10.0)	0.75 (0.09-6.58)
Hispanic	33 (0.1)	0.89 (0.62-1.28)	2 (6.1)	0.48 (0.11-2.17)
Non-Hispanic Black	142 (0.2)	1.15 (0.94-1.41)	8 (5.6)	0.50 (0.22-1.18)
Non-Hispanic White	361 (0.2)	1 [Reference]	37 (10.3)	1 [Reference]
Other^d^	7 (0.1)	0.51 (0.24-1.09)	0	NA^e^
Insurance				
Commercial	330 (0.2)	1 [Reference]	32 (9.7)	1 [Reference]
Medicare	160 (0.1)	0.60 (0.45-0.80)	13 (8.1)	1.50 (0.44-5.15)
Medicaid	57 (0.2)	0.92 (0.69-1.23)	3 (5.3)	0.56 (0.15-2.08)
Self-pay	6 (0.1)	0.63 (0.28-1.41)	0	NA^e^
Primary language				
English	552 (0.2)	1 [Reference]	48 (8.7)	1 [Reference]
Non-English	1 (<0.1)	0.06 (0.01-0.41)	0	NA^e^
Tobacco use				
Never or former	494 (0.2)	1 [Reference]	45 (9.1)	1 [Reference]
Current	59 (0.1)	0.70 (0.53-0.92)	3 (5.1)	0.41 (0.12-1.41)
Diabetes				
No	418 (0.2)	1 [Reference]	35 (8.4)	1 [Reference]
Yes	135 (0.1)	0.76 (0.62-0.93)	13 (9.6)	1.24 (0.60-2.57)
Depression				
No	431 (0.1)	1 [Reference]	34 (7.9)	1 [Reference]
Yes	122 (0.2)	1.30 (1.05-1.60)	14 (11.5)	1.51 (0.74-3.07)
Prescribed ≥2 BP medications				
No	392 (0.2)	1 [Reference]	34 (8.7)	1 [Reference]
Yes	161 (0.2)	1.24 (1.03-1.50)	14 (8.7)	0.93 (0.47-1.84)
Uncontrolled baseline BP				
No	399 (0.1)	1 [Reference]	31 (7.8)	1 [Reference]
Yes	154 (0.3)	1.82 (1.51-2.20)	17 (11.0)	1.72 (0.90-3.30)
No. of PCP visits				
1-2	199 (0.1)	1 [Reference]	14 (7.0)	1 [Reference]
≥3	354 (0.2)	1.48 (1.24-1.77)	34 (9.6)	1.63 (0.81-3.27)

Abbreviations: BP, blood pressure; NA, not applicable; OR, odds ratio; PCP, primary care physician.

^a^ Adjusted for age, sex, race and ethnicity, insurance, primary language, diabetes, tobacco use, depression, uncontrolled baseline BP, BP-lowering medications, and number of PCP visits.

^b^ Among 533 patients who received a BP monitoring order.

^c^ Among the subset of patients with hypertension who received an order for home BP readings from the care team during the study year.

^d^ Other includes American Indian or Alaska Native, Native Hawaiian, other Pacific Islander, and unknown.

^e^ None of the patients under other race or ethnicity, self-pay insurance, and non-English speakers shared BP readings with the care team, and therefore an OR could not be calculated.

### Associations With Clinical Characteristics

In the adjusted analysis, the number of PCP visits that the patient attended during the 1-year follow-up showed associations across all measures of engagement, with greater odds among patients who had 3 or more visits for any access (OR, 1.59; 95% CI, 1.56-1.61), access around PCP visit time (OR, 1.63; 95% CI, 1.61-1.66), frequent access (OR, 2.48; 95% CI, 2.45-2.52), and messaging (OR, 1.82; 95% CI, 1.80-1.85) compared with patients who had only 1 or 2 visits. Home blood pressure monitoring showed similar trends but was not statistically significant. In terms of comorbidities, patients with diabetes and depression were less likely to access patient portals in general (diabetes: OR, 0.90; 95% CI, 0.89-0.92; depression: OR, 0.96; 95% CI, 0.94-0.98) and around PCP visit time (diabetes: OR, 0.92; 95% CI, 0.91-0.94; depression: OR, 0.91; 95% CI, 0.90-0.93). However, the odds of engaging with messages (diabetes: OR, 1.07; 95% CI, 1.06-1.10; depression: OR, 1.15; 95% CI, 1.13-1.17) and sharing home blood pressure readings (diabetes: OR, 1.09; 95% CI, 1.07-1.10; depression: OR, 1.19; 95% CI, 1.17-1.22) were greater (Table 2).

## Discussion

In this cohort of more than 300 000 primary care patients with hypertension, 7 of 10 patients accessed the patient portal at least once between 2021 and 2022 but only 28.9% engaged in messages and 8.7% shared home blood pressure readings. The literature on quantifying patient portal engagement shows variable results depending on the patient population, how engagement is defined, and the time evaluated, with increased engagement in more recent years.^25^ An analysis by Chan et al^22^ reported that 53.4% of primary care patients accessed patient portals, of whom 74.5% were active users, defined based on accessing specific functionalities that require more active patient involvement. Sun et al^26^ reported that 32.9% of patients with diabetes attending outpatient clinics used patient portals.

Given the lack of a unified definition for engagement, we included several definitions beyond ever accessing the portal or frequency of access. Our results showed consistent findings of disparities in engagement regardless of the engagement definition used and after adjusting for several demographic and clinical characteristics. Although an access code for patient portal use is generated automatically for all patients attending this health care system, patients belonging to racial and ethnic minority groups were less likely to access the patient portal than non-Hispanic White patients. Patients whose preferred language is not English were also significantly less likely to engage with the patient portal, which is similar to findings in the study by Casillas et al.^27^ Although 100% of self-pay patients accessed the patient portal at least once, engagement was still low in this subgroup, which aligns with lack of engagement in other disadvantaged subgroups evaluated in this analysis. An explanation may be that these patients are using the self-pay function in the patient portal. Smoking, which is a social determinant of health–associated behavior, was also associated with less engagement with patient portals.

Our results extend the existing literature on the potential digital divide in patient portal engagement and highlight major disparities among patients treated for hypertension in primary care.^28,29^ The digital divide is the uneven distribution of access to, use of, and impact of information technologies between demographics and regions.^30^ Low patient portal use in vulnerable populations may result in intervention-generated inequity, which is a situation whereby an intervention inadvertently worsens existing health disparities instead of improving them.^31,32^ Research studies, interventions, and quality improvement initiatives in hypertension that are delivered via patient portals should be mindful of the disparities in engaging with patient portals. Efforts should be made and evaluated to reach all patients regardless of their race and ethnicity, insurance status, or preferred language. This is especially true for patients with hypertension because these subpopulations share the burden of clinical consequences.^7^

Our study did not explore the underlying reasons behind the digital divide, which may include education levels, low socioeconomic status, and lack of internet access.^25,33^ These characteristics likely mediate some of the associations observed in our analysis. In addition, physician and health care system characteristics also likely influence patient portal engagement.^30^ Self-reported survey data, such as the Health Information National Trends Study, found similar disparities in patient portal engagement. In that survey, patients who were non-Hispanic White were more likely to report being offered access to the patient portal (76.7%) compared with patients who are non-Hispanic Black (66.8%). This disparity highlights the importance of the care team actively discussing and offering the patient portal to patients and offering it equally regardless of race or ethnicity.^34^ In our analysis, the number of patients who received a self-monitored blood pressure order from the care team was too small to draw conclusions; however, data suggest that patients who are Hispanic or Latino and smokers were less likely to receive an order for self-monitored blood pressure. Future work should incorporate implementation science approaches and directly address the key role of physicians and staff in promoting more equitable patient portal use.^35^

Our study also sheds light on clinical characteristics related to hypertension that may be associated with patient portal engagement. More PCP visits were associated with greater patient portal engagement. This finding suggests that patients engaged with the patient portals are more engaged with their PCPs and have more in-clinic communication. Efforts should be made to engage patients with less than the guideline-recommended number of PCP visits (ie, <3 visits per year) with the patient portal as an alternative (backup) to in-person communications with the PCP.^25,30,33^

Further research is needed to understand how patient portal engagement is associated with reaching blood pressure control. An analysis from 2010 among patients with newly diagnosed hypertension showed an association between patient portal access (evaluated subjectively through a survey) and blood pressure control.^36^ However, the association disappeared after adjusting for potential confounders.^36^ More recently, a study evaluating associations between patient portal access and glycated hemoglobin control among patients with diabetes showed a clear link between accessing the portal and better control.^4^ More research exploring these associations among patients with hypertension is warranted.

### Limitations

A few limitations should be noted when interpreting our results. First, our data are limited to 1 health care system, and patients may seek care and use patient portals elsewhere. Second, unlike other studies that report on all the common functionalities of the patient portal, we focus our results on 2 functionalities that are relevant to patients with hypertension. Third, many factors may be associated with low patient portal engagement (eg, education level, income, and internet access); however, our analysis was limited to factors found within the EHR. Fourth, we were only able to evaluate patients’ engagement in home blood pressure monitoring via the patient portal–specific functionality. Patients may share their home blood pressure readings via the messaging functionality; however, this rate has been reported to be very low at 1.2% of patients only.^10^ Patients may also engage in home blood pressure monitoring outside patient portals, such as using automated blood pressure cuffs that transmit readings directly to the care team. However, few of these initiatives have been implemented in clinical settings given barriers related to the cost of the device to the patient and barriers to connecting these devices to the EHR.

## Conclusions

In this cohort study of patients with hypertension, two-thirds had accessed the patient portal at least once, with a median number of logins of 28 per year. Despite the observed high rates of engagement, clear disparities were observed in terms of race and ethnicity, insurance status, preferred language, and tobacco use. To overcome the digital divide and prevent further disparities in hypertension outcomes, it is important to further evaluate patient portal adoption among these subpopulations and identify ways of increasing their engagement.

## Notes

Supplement 1. **eAppendix.** Patient Flow Diagram

Supplement 2.
Data Sharing Statement
