# Supplementary material for: Disparities in Patient Portal Engagement Among Patients With Hypertension Treated in Primary Care
Source: JAMA Netw Open. 2024 May 15;7(5):e2411649. doi: 10.1001/jamanetworkopen.2024.11649 (PMC11096988; doi:10.1001/jamanetworkopen.2024.11649)
Supplement: Supplement 1. — eAppendix. Patient Flow Diagram [file jamanetwopen-e2411649-s001.pdf]

Supplementary Online Content

Khatib R, Glowacki N, Chang E, Lauffenburger J, Pletcher MJ, Siddiqi A. Disparities in patient portal engagement among patients with hypertension managed in primary care. *JAMA Netw Open*. 2024;7(5):e2411649. doi:10.1001/jamanetworkopen.2024.11649

eAppendix. Patient Flow Diagram

This supplementary material has been provided by the authors to give readers additional information about their work.

© 2024 Khatib R et al. *JAMA Network Open.*

eAppendix. Patient flow diagram

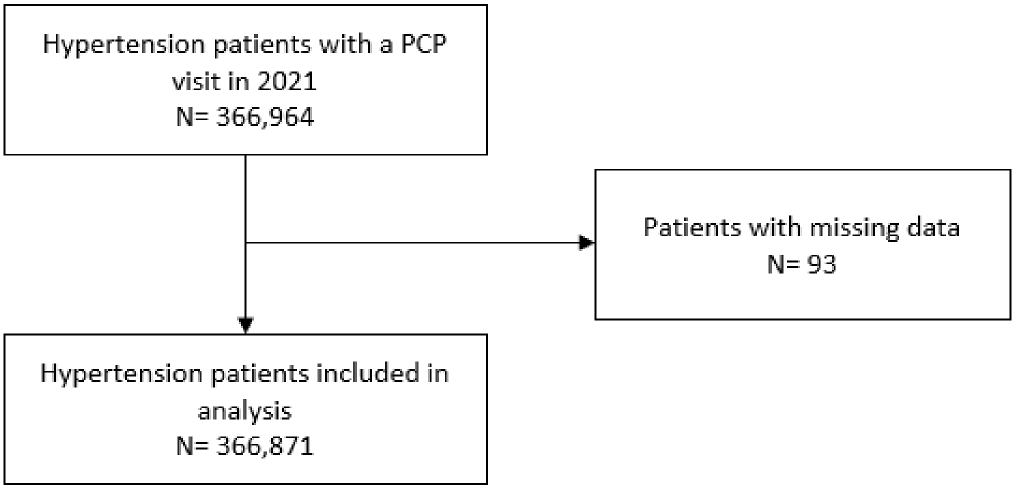

PCP visit: Primary Care Provider visit

© 2024 Khatib R et al. *JAMA Network Open.*
