# Supplementary material for: Disparities in Patient Portal Engagement Among Patients With Hypertension Treated in Primary Care
Source: JAMA Netw Open. 2024 May 15;7(5):e2411649. doi: 10.1001/jamanetworkopen.2024.11649 (PMC11096988; doi:10.1001/jamanetworkopen.2024.11649)
Supplement: Supplement 2. — Data Sharing Statement [file jamanetwopen-e2411649-s002.pdf]

Data Sharing Statement

Khatib. Disparities in Patient Portal Engagement Among Patients With Hypertension Managed in Primary Care. *JAMA Netw Open*. Published May 15, 2024.
doi:10.1001/jamanetworkopen.2024.11649

Data
Data available: No
